# Statins in patients with COVID-19: a retrospective cohort study in Iranian COVID-19 patients

**DOI:** 10.1186/s41231-021-00082-5

**Published:** 2021-01-25

**Authors:** Payam Peymani, Tania Dehesh, Farnaz Aligolighasemabadi, Mohammadamin Sadeghdoust, Katarzyna Kotfis, Mazaher Ahmadi, Parvaneh Mehrbod, Pooya Iranpour, Sanaz Dastghaib, Ahmad Nasimian, Amir Ravandi, Biniam Kidane, Naseer Ahmed, Pawan Sharma, Shahla Shojaei, Kamran Bagheri Lankarani, Andrzej Madej, Nima Rezaei, Tayyebeh Madrakian, Marek J. Los, Hagar Ibrahim Labouta, Pooneh Mokarram, Saeid Ghavami

**Affiliations:** 1 Health Policy Research Center, Institute of Health, Shiraz University of Medical Sciences, Shiraz, Iran; 2 Department of Epidemiology, Erasmus University Medical Center, Rotterdam, The Netherlands; 3 Autophagy Research Center, Shiraz University of Medical Sciences, Shiraz, Iran; 4 Department of Biostatistics and Epidemiology, School of Public Health, Kerman University of Medical Sciences, Kerman, Iran; 5 Department of Internal Medicine, Mashhad Medical Sciences Branch, Islamic Azad University, Mashhad, Iran; 6 Department of Anesthesiology, Intensive Therapy and Acute Intoxications, Pomeranian Medical University in Szczecin, 70-111 Szczecin, Poland; 7 Department of Analytical Chemistry, Faculty of Chemistry, Bu-Ali Sina University, Hamedan, Iran; 8 Influenza and Respiratory Viruses Department, Pasteur Institute of IRAN, Tehran, Iran; 9 Medical Imaging Research Center, Shiraz University of Medical Sciences, Shiraz, Iran; 10 Shiraz Endocrine and Metabolism Research Center, Namazee Hospital, Shiraz University of Medical Sciences, Shiraz, Iran; 11 Department of Clinical Biochemistry, Faculty of Medical Sciences, Tarbiat Modares University, Tehran, Iran; 12 Section of Cardiology, St. Boniface Hospital, University of Manitoba, Winnipeg, MB Canada; 13 Department of Surgery, University of Manitoba, Winnipeg, Manitoba Canada; 14 Department of Radiology, University of Manitoba, Winnipeg, Manitoba Canada; 15 Research Institute of Oncology and Hematology, Cancer Care Manitoba, University of Manitoba, Winnipeg, Canada; 16 Center for Translational Medicine, Thomas Jefferson University, Philadelphia, PA USA; 17 College of Pharmacy, Rady Faculty of Health Sciences, University of Manitoba, Winnipeg, Manitoba Canada; 18 Faculty of Medicine, Katowice School of Technology, 40-555 Katowice, Poland; 19 Research Center for Immunodeficiencies, Children’s Medical Center, Tehran University of Medical Sciences, Tehran, Iran; 20 Network of Immunity in Infection, Malignancy and Autoimmunity (NIIMA), Universal Scientific Education and Research Network (USERN), Tehran, Iran; 21 Biotechnology Center, Silesian University of Technology, 44-100 Gliwice, Poland; 22 Department of Biochemistry, School of Medicine, Shiraz University of Medical Sciences, Shiraz, Iran; 23 Department of Human Anatomy and Cell Science, Rady Faculty of Health Sciences, Max Rady College of Medicine, University of Manitoba, Winnipeg, Manitoba Canada

**Keywords:** COVID-19, SARS-CoV-2, Statins, Repurposing, Pleiotropic effects, Retrospective study

## Abstract

**Background:**

The coronavirus disease 2019 (COVID-19) pandemic caused by severe acute respiratory syndrome coronavirus 2 (SARS-CoV-2) infection has profoundly affected the lives of millions of people. To date, there is no approved vaccine or specific drug to prevent or treat COVID-19, while the infection is globally spreading at an alarming rate. Because the development of effective vaccines or novel drugs could take several months (if not years), repurposing existing drugs is considered a more efficient strategy that could save lives now. Statins constitute a class of lipid-lowering drugs with proven safety profiles and various known beneficial pleiotropic effects. Our previous investigations showed that statins have antiviral effects and are involved in the process of wound healing in the lung. This triggered us to evaluate if statin use reduces mortality in COVID-19 patients.

**Results:**

After initial recruitment of 459 patients with COVID-19 (Shiraz province, Iran) and careful consideration of the exclusion criteria, a total of 150 patients, of which 75 received statins, were included in our retrospective study. Cox proportional-hazards regression models were used to estimate the association between statin use and rate of death. After propensity score matching, we found that statin use appeared to be associated with a lower risk of morbidity [HR = 0.85, 95% CI = (0.02, 3.93), *P* = 0.762] and lower risk of death [(HR = 0.76; 95% CI = (0.16, 3.72), *P* = 0.735)]; however, these associations did not reach statistical significance. Furthermore, statin use reduced the chance of being subjected to mechanical ventilation [OR = 0.96, 95% CI = (0.61–2.99), *P* = 0.942] and patients on statins showed a more normal computed tomography (CT) scan result [OR = 0.41, 95% CI = (0.07–2.33), *P* = 0.312].

**Conclusions:**

Although we could not demonstrate a significant association between statin use and a reduction in mortality in patients with COVID19, we do feel that our results are promising and of clinical relevance and warrant the need for prospective randomized controlled trials and extensive retrospective studies to further evaluate and validate the potential beneficial effects of statin treatment on clinical symptoms and mortality rates associated with COVID-19.

## Background

The novel coronavirus, SARS-CoV-2, causing coronavirus disease 2019 (COVID-19) is a major health threat that was declared a global pandemic by the World Health Organization (WHO) in March 2020 [1–3]. Since the outbreak in December 2019, a considerable number of people has suffered from a severe acute respiratory infection that causes respiratory failure, the need for intubation, and prolonged mechanical ventilation [4]. Currently, the management of COVID-19 remains mostly supportive and depends on the severity of the disease, ranging from no support in asymptomatic patients to intubation and mechanical ventilation to manage acute respiratory distress syndrome (ARDS) in severe cases [5]. During the early stages of the pandemic, it was theorized that respiratory failure in COVID-19 may differ from typical ARDS [6]. These differences include the presence of good lung compliance, lack of pulmonary vasoconstriction, significant shunting and poor oxygenation, and concomitant features of thrombotic microangiopathy with endothelial dysfunction [7–9].

Apart from respiratory symptoms, most patients infected with SARS-CoV-2 have an increased risk of cardiovascular complications and thromboembolism, causing cerebrovascular events, deep vein thrombosis, or pulmonary embolism, further complicating the post-infection course. Patients suffering from COVID-19 frequently present with multi-morbidity (i.e., co-existence of multiple co-morbidities), including hypertension, cardiovascular disease, and diabetes. A retrospective analysis of 72,314 COVID-19 cases from China showed that patients with cardiovascular diseases exhibited significantly higher mortality (10.5% vs. 2.3%) [10]. The use of statins has been associated with a marked reduction in the risk of death from cardiovascular disease in the general population. Therefore, it has been proposed that statins through their pleiotropic modes of action, which include improvement of endothelial function, and anti-inflammatory and antithrombotic effects [11–13], may therapeutically benefit COVID-19 patients. A recent retrospective investigation on 13,981 patients with COVID-19 (Hubei Province, China), of which 1219 patients received statins, revealed that statin use was associated with reduced mortality [14].

Statins are conventionally used as cholesterol-lowering drugs, via inhibition of the mevalonate pathway, for first-line therapeutic prevention of atherosclerotic cardiovascular diseases [15, 16]. The discovery of their pleiotropic effects, independent of the cholesterol pathway, further attracted attention to this class of drugs [17]. The decreased mortality rate from cardiovascular diseases following statin consumption was attributed to both the lipid-lowering effect and enhancement of vascular endothelial function [18–20]. The latter statin-effect has also been observed in other patient populations, including diabetics [21, 22] and women with polycystic ovary syndrome [23]. Various mechanisms have been reported for the statin-mediated improvement of endothelial function and include reducing remnant lipoproteins [24], modulating the high mobility group box 1/toll-like receptor 4(HMGB1/TLR4) pathway [25], inhibiting miR-133a expression [26], inducing nitric oxide release [27], affecting Kruppel-like factor 2 signaling [28], and decreasing the level of acute-phase proteins (like C-reactive protein) [29]; of note, this last mechanism is also involved in the anti-inflammatory effects of statins [29, 30]. Due to their anti-inflammatory properties, statins showed beneficial effects in hypertensive patients with normal cholesterol levels [19, 20]. Several studies have identified the activation of autophagy as one of the primary molecular mechanisms underpinning the anti-inflammatory effects of statins [31–33].

In the current retrospective study, we investigated whether statin use is associated with improved survival and clinical outcome in COVID-19 patients who were hospitalized in the Ali Asghar Hospital, Iran.

## Methods

### Patient recruitment

**Fig. 1:**
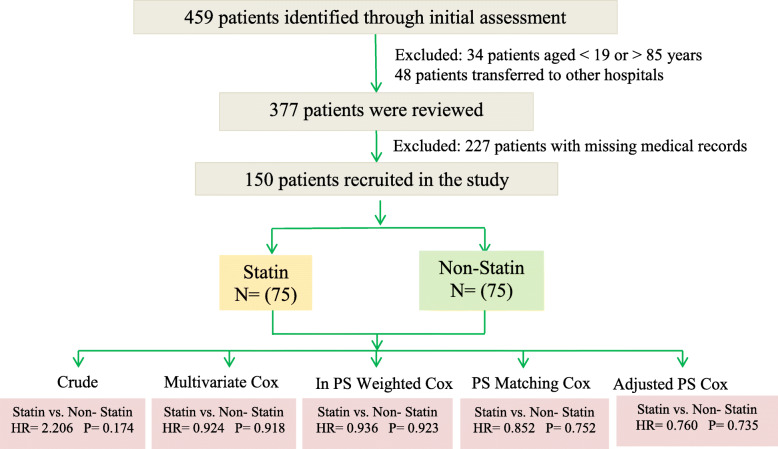
Flow-chart of patient enrolment and summary of the inclusion and exclusion criteria. HR: Hazard Ratio; P: P-value; P S: Propensity Score; In PS: Invers Propensity Score

This was a retrospective study involving a population of COVID-19 patients (ethics approval: IR.SUMS.REC.1399.151, Shiraz University of Medical Sciences) treated in a single tertiary hospital in Shiraz, Iran (ALIASGHAR Hospital), between March 1st and May 30th, 2020. We specifically focused on those patients who were discharged or had died by the end of May 2020 (Fig. 1). Hospitalized patients with the following inclusion criteria were evaluated: COVID-19 pneumonia confirmed as per WHO criteria, including a positive SARS-CoV-2 polymerase chain reaction (PCR) test of respiratory (nasopharynx, oropharynx) specimens, and evidenced by chest X-ray or chest computed tomography (CT) scan. The CT images were reviewed by an expert radiologist who was blind to the clinical information. Each CT scan was scrutinized for the presence of important radiologic features, including ground-glass opacity (GGO), consolidation, pleural effusion, septal thickening, and lymphadenopathy. The Radiological Society of North America Expert Consensus guidelines were implemented for the diagnosis of positive cases [34]. The peripheral bilateral GGOs, GGOs of rounded morphology, reverse halo sign, and other indications of organizing pneumonia were regarded as typical for COVID-19 pneumonia. A radiologic severity score was applied in case of abnormal chest CT findings. Patients were also required to meet the following oxygenation inclusion criteria: SPO_2_ ≤ 93% or PaO_2_/FiO_2_ < 300 mmHg < 300 mmHg. The exclusion criteria were: HIV, hepatitis B or C, influenza virus, or active tuberculosis infection; suspected active bacterial, fungal, or any other infections besides COVID-19; age < 19 and > 85 years old. In addition, patients with autoimmune or neoplastic diseases were excluded as they were using immune-modulators and/or anti-inflammatory medications or participated in drug clinical trials.

Initially, a total of 459 patients were considered eligible for inclusion. Of these patients, 34 were excluded in the primary screening since they did not meet the age criteria (Fig. 1). During the first phase of our investigation, 48 patients were transferred to other hospitals and excluded from the study because of the inability to follow-up. An additional 227 patients were excluded because important aspects of their medical records were missing (Fig. 1). Ultimately, a total of 150 laboratory-confirmed COVID-19 patients were included in this study, of which 75 had a history of statin therapy and still used statin at the time of hospitalization. Patients using statins were matched with those that did not according to specific confounders, such as duration of the disease, COVID-19 medication, stage of the disease, history of the underlying disease, and age. Demographic, clinical treatment, and laboratory data (including serial samples for viral RNA detection) were extracted from the electronic medical records. All data were checked by the physicians and a researcher adjudicating any differences in the interpretation by the primary radiologists. After data collection, univariable logistic regression was performed to screen for predictors, after which multivariable logistic regression was conducted to evaluate the effects of statin therapy and other predictors on patient recovery.

### Statistical analysis

Cox proportional-hazards regression models were used to estimate the association between statin use and risk of death. The proportional hazard (PH) assumption was checked for all predictors with the Schoenfeld residuals analysis test [35] before model building. The PH assumption is very important to Cox regression; it means that the ratio of the hazards for any two individuals is constant over time. For example, if gender is one of the predictors in the Cox model and the hazard of death for men is twice as high as for women, this must be constant over time. It dictates that time-varying predictors cannot enter the PH Cox model [35].

First, univariate Cox regression was conducted for each predictor. Variables that had a significant association with the hazard of death in this analysis were subsequently entered into the multivariate Cox regression.

To adjust for the effects of confounders and estimate the pure association between statin consumption and time until death, we implemented four strategies: multivariate Cox regression, Weighted PH Cox, PH Cox after propensity score matching, and PH Cox adjusted with propensity score. In our study, individual matching was very difficult or even impossible because protecting the patient’s life is a priority and all physicians want to prevent their patients from dying; therefore, physicians often prescribe several antibiotics and antivirals simultaneously. Consequently, there were several confounders, such as drugs and clinical-, laboratory-, and CT- characteristics.

A confounder is a variable that simultaneously has an association with the exposure and the outcome. In the statistical analysis of observational data, there is no randomization of the participants in treatment groups. Therefore, the participant populations are not homogeneous based on their characteristics and clinical variables. For example, in our study, patients in the statin and non-statin groups were not subjected to the exact same amount or type of antivirals, antibiotics and/or other drugs. Multiple drug-related, laboratory, and characteristic variables were different between these groups; such heterogeneous variables led to the bias estimation of the statin effect.

We used propensity score methods to minimize the effects of confounding factors. The propensity score analysis is a statistical technique that estimates the pure effect of treatment and removes or reduces the effect of other confounding variables. By using this method, the effects of all confounding variables are summarized into a score (the propensity score). The individual propensities were estimated with the use of a multivariable logistic regression model that included all confounding covariates; statin use was considered the outcome in this approach.

We performed three adjustment procedures with respect to the propensity score. First, the individual propensity scores were inversed to calculate the inverse propensity score weights [36]. Analysis using the Weighted Cox regression model was subsequently conducted with these weights (Weighted PH Cox with inverse propensity score as weight). Second, since each individual has a propensity score, subjects from the two groups (statin vs no statin) could be matched based on their scores [37]. In the propensity score matching analysis, the nearest-neighbor method was applied to create the best matches (PH Cox after propensity score matching). In the third adjustment procedure, the propensity score was entered in the multivariate Cox regression as an additional covariate (PH Cox adjusted with propensity score). For the secondary analysis, we used logistic regression and the outcome was changed to CT result and mechanical ventilation. Multiple imputations were used to handle the missing data [38]. All statistical analyses were performed using R software, version 4.02 (R Project for Statistical Computing).

## Results

For the 150 recruited participants with COVID-19 that met the inclusion criteria, the mean duration of hospitalization was 6.84 ± 4.35 days. The majority of patients was retired (29.7%) and/or stayed occupied doing housework (31.8%). The mean age and BMI were 54.69 ± 4.35 years and 26.27 ± 3.68, respectively. Follow-up with these patients started at admission and continued for 27 days. Over this period, 19 patients died from COVID19, whereas the other 131 patients recovered and were discharged from the hospital. Three different types of statin were used among the 75 patients on statins: atorvastatin (94.7%), rosuvastatin (2.7%), and simvastatin (2.7%). In the statin group, 14% of patients had been using statins for over 5 years, 22% for 1–5 years, and the remaining 64% for less than 1 year before hospital admission. The mean time of statin use during hospitalization was 7.37 ± 4.84 (SD) days.

Patients that used statins (40 mg daily) because of hyperlipidemia were older (65.61 vs. 58.77 years of age, *P* < 0.001) and had a higher prevalence of comorbidities, including hypertension (48% vs. 10.7%, *P* < 0.001), type 2 diabetes (33.3% vs. 9.3%, *P* < 0.001), cardiovascular disease (2.7% vs. 0%, *P* < 0.001), cerebrovascular diseases (10.7% vs. 1.3%, *P* < 0.001), and chronic kidney diseases (25% vs. 8%, *P* < 0.001), than those without statin therapy. On average, the statin group exhibited significantly increased leukocyte polymorphonuclear (PMN) percentages (4.99 ± 2.55 vs. 4.94 ± 3.04) and reduced lymphocyte counts (1.32 ± 0.84 vs. 1.79 ± 0.86) as compared to the non-statin group (*P* = 0.01) (Table 1).

**Table 1:** Characteristics and comparison of patient groups (statin vs no statin), before and after propensity score matching

Parameters	Unmatched			Matched		
Characteristics variables	Statin (75 patients)	No statin (75 patients)	*P*-value	Statin (75 patients)	No statin (75 patients)	*P*-value
Age [years]	65.61 ± 13.22	58.77 ± 16.01	**< 0.001**	63.59 ± 13.18	61.72 ± 15.83	0.433
BMI	25.92 ± 4.02	26.61 ± 3.26	0.270	25.81 ± 3.91	25.46 ± 3.16	0.545
Gender, n (%)						
Male, n (%)	47 (62.7)	40 (53.3)	0.247	45 (60)	43 (57.3)	0.740
Female, n (%)	28 (37.3)	35 (46.7)		30 (40)	32 (42.7)	
Addiction status, n (%)	14 (18.7)	1 (1.3)	**0.001**	11 (14.66)	7 (9.33)	0.315
**Triage vital signs**						
Heart rate, beats per min	91.04 ± 18.13	94.20 ± 14.56	0.241	91.02 ± 18.11	92.12 ± 11.82	0.660
Respiratory rate, breathes per min	19.56 ± 3.85	19.44 ± 2.45	0.970	19.52 ± 2.31	19.42 ± 2.22	0.746
Systolic blood pressure (mm Hg)	130.61 ± 23.23	124.28 ± 17.17	0.246	129.58 ± 22.19	127.55 ± 11.1	0.448
Diastolic blood pressure (mm Hg)	78.01 ± 13.23	78.52 ± 11.56	0.841	78.01 ± 12.98	78.08 ± 11.58	0.972
Body temperature (°C)	37.45 ± 1.14	37.41 ± 1.04	0.787	37.31 ± 1.09	37.22 ± 1.02	0.602
Oxygen saturation (SpO2)	87.65 ± 6.52	91.74 ± 4.60	**< 0.001**	88.92 ± 5.88	90.23 ± 3.82	0.108
**Sign and symptoms**						
Chest pain, n (%)	4 (5.3)	8 (10.3)	0.367	6	7	> 0.999
Dyspnea, n (%)	62 (82.7)	49 (65.3)	**0.025**	58	52	0.268
Cough, n (%)	51 (68)	58 (77.3)	0.309	53	51	0.068
Headache, n (%)	13 (17.3)	19 (25.3)	0.319	15	17	0.690
**Comorbidities**						
Hypertension, n (%)	36 (48)	8 (10.7)	**< 0.001**	31	12	**0.001**
Diabetes, n (%)	25 (33.3)	7 (9.3)	**< 0.001**	21	10	0.524
Cardiovascular disease, n (%)	2 (2.7)	0 (0)	**< 0.001**	2	1	> 0.999
Respiratory disease, n (%)	9 (12)	10 (13.3)	> 0.999	8	9	0.229
Chronic kidney disease, n (%)	19 (25)	6 (8)	**0.004**	15	8	0.113
**Laboratory measures**						
PMN percent (%)	4.99 ± 2.55	4.94 ± 3.04	**0.010**	4.94 ± 2.31	4.93 ± 2.82	0.981
Lymphocyte count in serum	1.32 ± 0.84	1.79 ± 0.86	**0.001**	1.38 ± 0.71	1.61 ± 0.74	0.054
Prothrombin time (PT), (s)	35 ± 17.43	15.70 ± 3.34	**< 0.001**	31.01 ± 11.07	19.36 ± 2.99	< 0.001
International Normalized Ratio (INR)	1.59 ± 0.62	1.54 ± 0.55	0.602	1.58 ± 0.61	1.56 ± 0.45	0.819
C-reactive protein (CRP), (ml/dl)	78 ± 30.34	24.82 ± 21.46	0.112			
**Antibiotics therapy**						
Vancomycin, n (%)	33 (44)	35 (46.7)	0.743	33	34	0.870
Linezolid, n (%)	31 (41.3)	12 (16)	**0.001**	28	16	**0.031**
Ceftriaxone, n (%)	27 (36)	30 (40)	0.614	28	29	0.866
Azithromycin, n (%)	17 (22.7)	21 (28)	0.453	19	20	0.852
**Antiviral therapy**						
Ribavirin, n (%)	8 (10.7)	10 (13.3)	0.615	8	9	0.797
Lopinavir/Ritonavir, n (%)	40 (53.3)	36 (48)	0.514	39	37	0.744
Oseltamivir, n (%)	42 (56)	37 (49.3)	0.414	41	38	0.624
**Other therapy**						
Hydroxychloroquine, n (%)	67 (89.3)	63 (84)	0.377	65	65	> 0.999
Glucocorticoids, n (%)	10 (13.3)	4 (5.3)	0.092	9	6	0.414
Salbutamol, n (%)	25 (33.3)	15 (20)	0.065	21	18	0.577
Seroflo, n (%)	12 (16)	12 (16)	> 0.999	12	12	> 0.999
**Respiratory support**						
Mechanical ventilation, n (%)	30 (40)	16 (21.3)	**0.013**	27	19	0.464

*BMI* body mass index; *P*-values were calculated by Mann-Whitney U test for non-normally distributed continuous variables and Fisher’s exact test or χ^2^ test for the categorical variables; *P*-value less than 0.05 was considered significant

The baseline patient characteristics are shown in Table 1, both in the unmatched and propensity score-matched samples. Before matching individuals from the two groups (statin vs non-statin) based on the propensity score, some variables showed different distribution; however, after matching, the majority showed a similar distribution in both groups. In the unmatched samples, statin exposure differed according to age, addiction status, hypertension, diabetes, cardiovascular disease, chronic kidney disease, dyspnea, oxygen saturation (SpO_2_), PMN percentage, lymphocyte count, prothrombin time, linezolid treatment, and mechanical ventilation (at least *P* < 0.05 for all).

**Fig. 2:**
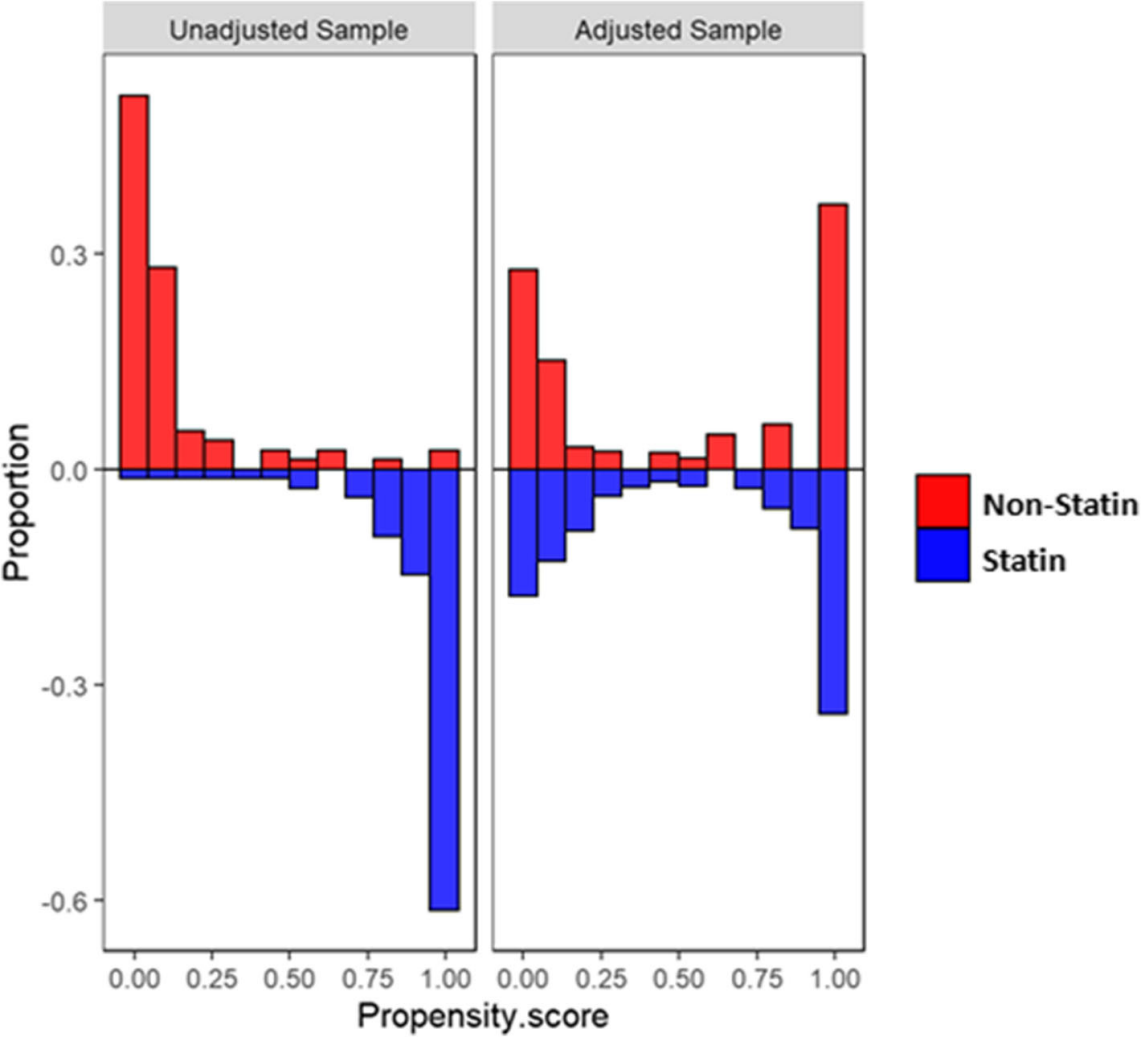
Histogram and density distribution plots of the estimated propensity scores, before and after propensity score adjustment in statin and non-statin patient groups. The left side of the figure (unadjusted) shows that the non-statin group had more density on the right side of the plot versus the statin group that had more density on the left side of the plot. After adjustment (right side of the figure), both groups displayed similar densities. The results show that the two groups became more similar when considering mutual confounders that may affect the association between the main exposure variable (statins) and survival time. The C-statistic of the propensity score model was 0.75

The distribution of the estimated propensity scores among statin and non-statin patients is shown in Fig. 2. As can be clearly observed, the histogram and density distribution of the estimated propensity scores were more balanced after adjustment. This shows that the two groups became more similar when considering mutual confounders that may affect the association between the main exposure variable (statins) and survival time. The C-statistic of the propensity score model was 0.75.

**Fig. 3:**
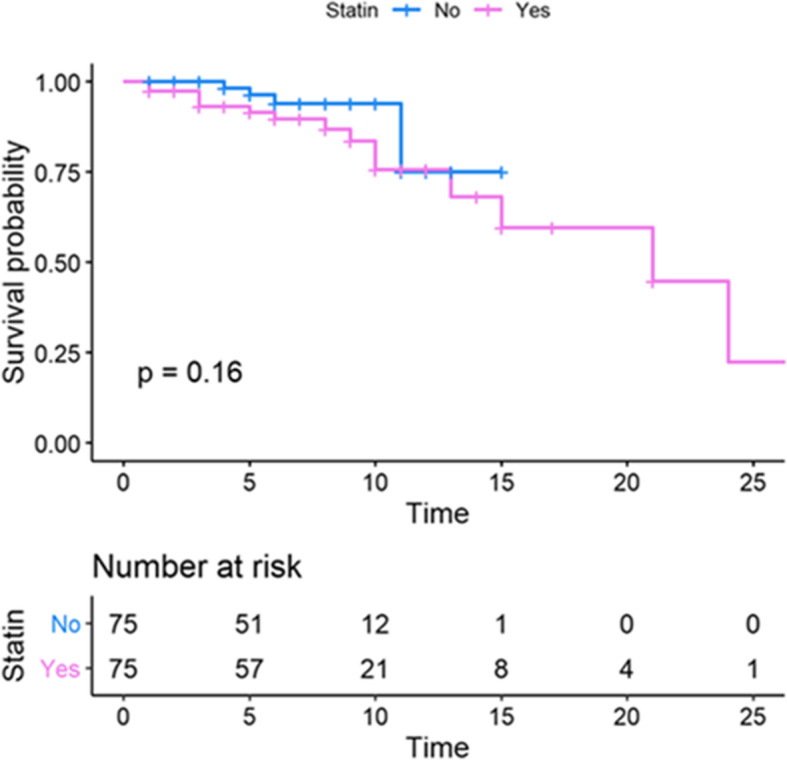
Kaplan-Meier plot comparing the survival time (in days) between the statin and non-statin groups. This plot suggests that before any adjustment (naive data) mortality was lower in the statin than the non-statin group. However, the *P*-value of the log-rank test (*P* = 0.16) did not indicate any significant difference

**Fig. 4:**
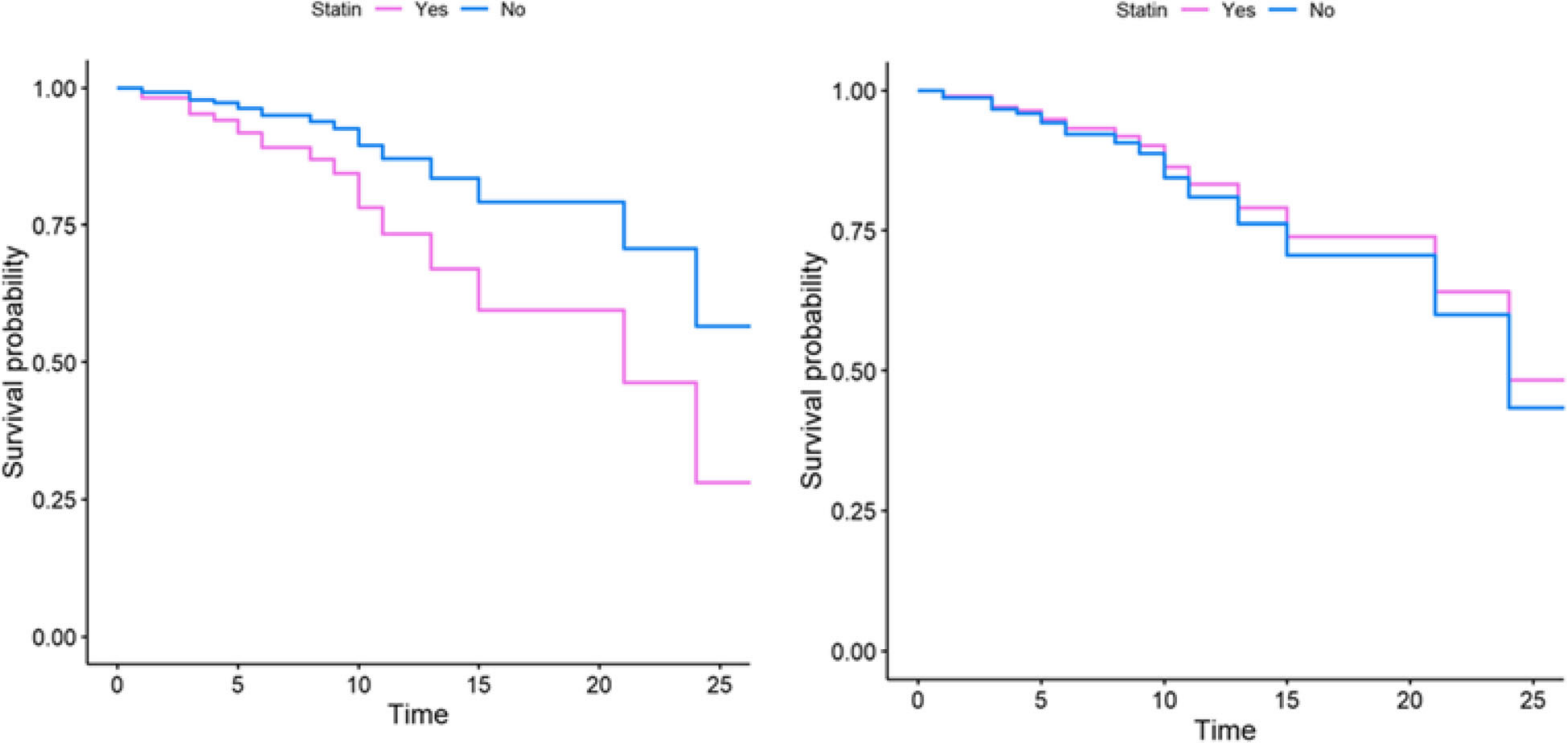
Crude survival and propensity score-adjusted Cox plots comparing the survival time (in days) between the statin and non-statin groups. The plots highlight the difference between crude and propensity score-adjusted survival Cox regression plots for the statin and non-statin patient populations. These plots suggest that before adjustment (left plot), the statin group displayed a lower survival time, whereas after adjustment (right plot), statin users showed a higher survival probability; however, neither of these differences was significant

Figure 3 shows the Kaplan–Meier curve of the statin and non-statin groups. This plot suggested that before any adjustment (naive data) the statin group had a lower survival compared to the non-statin group; however, this difference was not statistically significant. (*P* = 0.16). The proportional hazard (PH) assumption was checked and approved for all the characteristic variables before conducting PH Cox model analyses (not shown). The crude and adjusted hazard ratios (HRs) for the association of characteristic variables on survival time are shown in Table 2. In the crude, unadjusted analysis, patients on statins seemed more likely to have a death event but this did not reach significance [HR = 2.21, 95% CI = (0.71,6.90), *P* = 0.174]. The variables that showed a significant association in the univariate model (crude) were entered into the multivariate Cox regression model for adjustment purposes. In the multivariate Cox model, only mechanical ventilation had a significant effect on survival. Interestingly, the adjusted HR in the multivariate model that included mechanical ventilation was associated with an apparent lower risk of mortality in COVID19 patients on statins compared to non-statin users [HR = 0.92, 95% CI = (0.21,4.16), *P* = 0.918]; although a promising observation, this association was not statistically significant. Figure 4 clarifies and highlights the difference between crude and propensity score-adjusted Cox regression plots for patient survival in the statin and non-statin groups. The results of five distinct Cox models with different adjustments are outlined in Table 3. No association was found between statin use and reduced mortality survival in the Cox analysis with inverse propensity score weighting (weighted Cox regression) [HR = 0.94; 95% CI = (0.22, 4.17), *P* = 0.923**]**. Based on a Cox model after propensity score matching, the HR for the statin group appeared to be associated with a lower risk of mortality [HR = 0.85, 95% CI = (0.02,3.93), *P* = 0.762]. Similarly, additional propensity score analysis results (Cox regression with propensity score adjustment, model 5) also revealed that statin use was potentially associated with a decreased hazard of death [(HR = 0.76; 95% CI (0.16, 3.72), *P* = 0.735)]. Although these associations did not reach statistical significance, they do indicate that statin use might increase survival of COVID19 patients, and that future prospective clinical trials and extensive retrospective studies are warranted to validate the potential therapeutic potential of statins in the amelioration of clinical symptoms and mortality rate associated with COVID19.

**Table 2:** Data analysis using the crude and adjusted Cox regression models

Parameters	Crude			Adjusted		
Variables	HR	95% C.I for HR	***P***-value	HR	95% C.I for HR	***P***-value
Age	1.02	0.97–1.05	0.294	–	–	–
BMI	1.05	0.92–1.19	0.503	–	–	–
Gender (men vs. women)	1.16	0.45–3.01	0.760	–	–	–
Smoking vs. none	0.83	0.26–2.67	0.752	–	–	–
Addiction vs. none	1.63	0.45–5.93	0.462	–	–	–
**Triage vital signs**						
Heart rate, beats per min	0.98	0.95–1.02	0.066	–	–	–
Respiratory rate, breathes per min	1.05	0.97–1.14	0.263	–	–	–
Systolic blood pressure (mm Hg)	0.96	0.94–0.99	**0.005**	1.01	0.98–1.05	0.507
Diastolic blood pressure (mm Hg)	0.91	0.88–0.95	**< 0.001**	0.95	0.88–1.04	0.244
Body temperature, Celsius degrees	1.06	0.70–1.61	0.781	–	–	–
Oxygen saturation (SpO2)	0.89	0.84–0.95	**< 0.001**	0.97	0.86–1.10	0.637
**Sign and symptoms**						
Chest pain vs. none	0.51	0.06–4.25	0.531	–	–	–
Dyspnea vs. none	2.49	0.57–10.98	0.226	–	–	–
Cough vs. none	0.49	0.19–1.27	0.142	–	–	–
Headache vs. none	1.60	0.56–4.58	0.379	–	–	–
**Comorbidities**						
Hypertension	2.27	0.89–5.75	0.086	–	–	–
Diabetes	1.55	0.59–4.01	0.369	–	–	–
Cardiovascular disease	2.38	0.93–6.06	0.070	–	–	–
Respiratory disease	1.19	0.34–4.15	0.787	–	–	–
Chronic kidney disease	4.20	1.66–10.67	**0.003**	2.21	0.49–9.89	0.302
**Laboratory measures**						
PMN percent	1.04	1.05–1.16	**< 0.001**	1.04	0.97–1.13	0.262
Lymphocyte count	0.88	0.81–0.95	**0.001**	1.04	0.93–1.13	0.486
Prothrombin time (PT)	0.99	0.99–1.01	0.755	–	–	–
International Normalized Ratio (INR)	1.87	1.21–2.88	**0.005**	1.43	0.58–3.55	0.441
C-reactive protein (CRP), (ml/dl)	0.99	0.97–1.02	0.995	–	–	–
Serum Creatinine	1.11	1.01–1.22	**0.035**	1.08	0.88–1.33	0.461
Hematocrit	0.85	0.78–0.93	**0.001**	0.91	0.79–1.04	0.774
**Antibiotics therapy**						
Vancomycin vs. none	1.30	0.49–3.43	0.591	–	–	–
Linezolid vs. none	0.81	0.31–2.17	0.679	–	–	–
Ceftriaxone vs. none	0.81	0.32–2.07	0.662	–	–	–
Azithromycin vs. none	0.22	0.03–1.63	0.137	–	–	–
**Antiviral therapy**						
Ribavirin vs. none	1.04	0.30–3.62	0.948	–	–	–
Lopinavir/Ritonavir vs. none	1.33	0.51–3.48	0.562	–	–	–
**Other therapy**						
Glucocorticoids vs. none	3.17	1.16–8.66	**0.025**	0.67	0.12–3.82	0.649
ARB vs. none	2.49	0.89–7.01	0.082			
Statin vs. none	2.21	0.71–6.90	0.174	0.92	0.21–4.16	0.918
Salbutamol vs. none	0.53	0.18–1.52	0.236	–	–	–
Seroflo vs. none	0.35	0.08–1.56	0.167	–	–	–
**Respiratory support**						
Mechanical ventilation vs. none	11.01	2.48–18.85	**0.002**	6.27	1.03–11.27	**0.047**

*HR* hazard ratio, *C.I* confidence interval, *BMI* body mass index, *ARB* angiotensin receptor blocker

*P*-value less than 0.05 was considered significant

**Table 3:** The effect of statin use on COVID-19 patient survival as assessed by the crude, multivariable and propensity score analysis

Cox models	Model	HR	95% C.I for HR	***P***-value
Crude	1	2.21	0.71–6.90	0.174
Multivariate Cox	2	0.92	0.21–4.16	0.918
Weighted PH Cox (IPS as weight)	3	0.94	0.22–4.17	0.923
PH Cox after PS Matching	4	0.85	0.02–3.93	0.762
PH Cox adjusted with PS	5	0.76	0.16–3.72	0.735

*HR* hazard ratio, *CI* confidence interval, *PH* proportional hazard, *PS* propensity score, *IPS* inverse propensity score

As a secondary outcome, we used logistic regression to analyze the association of statin use with the incidence of invasive mechanical ventilation and abnormal CT results in COVID19 patients. After adjusting with the propensity score, statin consumption was associated with a lower chance of being subjected to mechanical ventilation [OR = 0.96, 95% CI = (0.61–2.99), *P* = 0.942]. In addition, patients in the statin group appeared less likely to present with abnormal CT results [OR = 0.41, 95% CI = (0.07–2.33), *P* = 0.312] (Table 4).

**Table 4:** The results of the crude and propensity score adjusted logistic regression for the secondary outcome

Logistic regression models	Crude			Adjusted with PS		
Secondary outcome	OR	95% CI for OR	*P*-value	OR	95% C.I for OR	*P*-value
CT result (abnormal vs. normal)	1.77	0.61–5.14	0.295	0.41	0.07–2.33	0.312
Mechanical ventilation vs. none	2.46	1.19–5.05	**0.014**	0.96	0.61–2.49	0.942

*OR* odds ratio, *CI* confidence interval, *PS* propensity score

## Discussion

The results on statin use and its potential benefits for COVID-19 patients are controversial. In a recent meta-analysis, it was reported that “statin use did not improve in-hospital outcomes of COVID-19 infections” [39]; this analysis included 9 studies with a total of 3449 patients. The results showed that statin use did not improve the outcome severity [OR = 1.64, 95% CI: (0.51–5.23), *P* = 0.41, I2 = 93%, random-effect modelling] or the mortality rate associated with COVID-19 infection [OR 0.78, 95% CI: (0.50–1.21), *p* = 0.26, I2 = 0%, fixed-effect modelling] [39]. This finding might be due to the statistical analysis method used. In an investigation on the use of statins in chronic renal disease patients with COVID-19, it was shown that statins reduced “the risk of neutrophilia [OR-0.10, 95% CI: (0.01-0.69)]” but did not affect the mortality in these patients [40]. Conversely, a relatively large retrospective study on 13,981 patients with COVID-19 (Hubei Province, China) revealed that statin use decreased all-cause mortality from 9.4 to 5.2% (a total of 1219 patients received statins) [14]. Furthermore, a recent investigation showed that statin use decreased the in-hospital mortality in COVID-19 patients with diabetes [41]. It has also been reported that for patients using statin during hospitalization the risk of ICU admission is reduced when compared to non-statin users [42], and that statin use decreased the risk of invasive mechanical ventilation in COVID-19 patients [43].

Based on our overall results, we feel that there is an indication that the use of statins may be linked to a lower risk of COVID19 mortality, even though this association did not reach statistical significance [HR = 0.924, 95% CI = (0.205, 4.157), *P* = 0.918]. In addition, statins significantly reduced the need for mechanical ventilation and improved lung CT in COVID-19 patients. Although promising and clinically relevant, our observed associations between statin use and survival of COVID19 patients were not statistically significant (likely due to impaired power of our analysis as a result of the many patients that had to be excluded) and need to be validated in prospective clinical trials and extensive retrospective studies. Considering the various documented pleotropic effects of statins and the results presented here, we anticipate positive effects of statin use on the clinical outcomes of COVID-19 patients. In a recent investigation comprising 3654 individuals in Germany, it was demonstrated that using statins was associated with asymptomatic COVID-19; accordingly, it was postulated that statins might promote SARS-CoV-2 transmission by increasing the number of asymptomatic COVID-19 patients [44]. In contrast, a recent observational multi-center study in Italy indicated that statin use was associated with an increased risk of more severe COVID-19 [45], whereas another recent report suggested that statins did not affect mortality at all in COVID-19 patients (4842 subjects) [46].

Pleiotropic effects of statins have been reported in several malignancies, especially respiratory infections [47, 48], acute lung injury [49], pulmonary hypertension [50], community-acquired pneumonia, chronic obstructive pulmonary disease [51], and interstitial lung disease [52]. Statins have shown various beneficial anti-inflammatory and immunomodulatory properties by affecting intracellular signaling pathways [53, 54] that are independent of their lipid-lowering ability [55]. Furthermore, antiviral effects against the influenza virus have been demonstrated in several in vitro and in vivo studies [56–59]. Statins modulate the antiviral response in human bronchial and epithelial cells, which constitute the first line of defense against invading pathogens [60], and significantly reduce the production of pro-inflammatory cytokines, such as TNF-훼 and IL-6, in Crandell feline kidney cells infected with H1N1 [61]. In primary normal human bronchial epithelial cells (NHBE) and the human type II pneumocyte cell line A549, simvastatin attenuated viral dsRNA-induced AKT phosphorylation, STAT3 activation, and subsequent production of RANTES [62]. In another study, simvastatin was shown to reduce the replication of H1N1 by blocking RhoA membrane localization, actin filament condensation, Rab protein expression during endocytosis, and LC3-II protein localization [57].

The effects of statins on the *Coronaviridae* family are still under-studied. Earlier in 2015, it was proposed that a high dose of atorvastatin may work against MERS-CoV infections by decreasing inflammatory cytokines, and that although statins may not be very effective in late-stage patients, timely use could be vital for the survival of MERS-CoV infected subjects [63]. Since some investigations had confirmed the association of statin therapy with a reduction in cardiovascular outcomes and mortality in patients infected with influenza, it was also suggested that COVID19 patients, with severe damage to lung tissue caused by a cytokine storm of inflammatory mediators, continue statin therapy for its potential clinical benefit [64].

Several studies have provided some more mechanistic perspectives on the action of statins in relation to SARS-CoV-2 infection. An in silico docking study highlighted that statins might be efficient inhibitors of the SARS-CoV-2 main protease [65]. Moreover, selective statins (fluvastatin was more efficient than other statins) reduced SARS-CoV-2 cell entry and inhibited infection of human respiratory epithelial cells in vitro by either the low pathogenic (coronavirus 229E) or highly pathogenic (2019-nCoV) coronavirus [66]. This study also reported that statin therapy did not cause any additional risk to patients and that some statins may have a mild beneficial effect on COVID-19 outcome [66].

Autophagy is an important cellular mechanism against different types of stress, including viral infection [3, 67, 68]. SARS-CoV-2 infection likely changes autophagy flux in the infected cells and hijacks it for its replication [3]. The clinical effects of statins described to date have in part been attributed to their impact on cellular autophagy. Indeed, several studies have reported the involvement of the autophagy pathway as a mechanism for the protective effects of statins in the human lung [69, 70]. Using airway mesenchymal cells, we demonstrated the potential for statins to have beneficial effects in obstructive airways diseases by induction of autophagy via upregulation of p53 [71] as well as through their effects on other pathways, such as the unfolded protein response [72]. Using a mouse model of asthma, simvastatin was shown to ameliorate key asthmatic symptoms (airway remodeling and inflammation) in the lungs via autophagy augmentation [33]. Therefore, it is conceivable that statins may target SARS-CoV-2 infection in lung epithelial cells through autophagy(−associated) signaling.

Statins have shown modulatory effects on angiotensin-converting enzyme 2 (ACE2) expression. Qi and co-workers were the first to study the expression of ACE2 in cardiomyocytes of rats with cardiac hypertrophy and reported a decreased expression of both ACE2 mRNA and protein levels in response to 4 weeks of treatment with 5 mg/kg/day atorvastatin [73]. Later, similar effects on aortic arteries in vascular balloon injured rats treated with the same doses of rosuvastatin for 2 to 4 weeks were demonstrated [74]. However, other studies presented conflicting observations. In a cellular model of vascular smooth muscle cell hypertrophy, atorvastatin recovered the molecular changes induced by TNF-α and increased ACE2 mRNA expression [75]. Using a rabbit model of atherosclerosis, an association between atherosclerosis and a reduced level of ACE2 in both renal and cardiac tissue was established; ACE2 levels were replenished following 3 weeks of atorvastatin administration [76]. Since the emergence of the significance of ACE2 expression in diabetes [77], scientists have focused on the potential role of statins in diabetes. Aguilar and colleagues showed that atorvastatin increased ACE2 mRNA in cardiomyocytes of diabetic cardiomyopathic rats, which was associated with a reduction in fibrosis and hypertrophy of the left ventricles [78]. These effects were attributed to the recovered ratio of ACE/ACE2 rather than an increased level of ACE2 [78]. Further studies in diabetic rats confirmed that statin administration increased ACE2 mRNA in cardiac tissue from insulin-controlled groups, although this was not accompanied by significant effects on cardiac fibrosis and reactive oxygen species generation [79, 80]. In our current report, 33% of COVID19 cases that used statins were diabetic. Although diabetes is among the comorbidity factors for COVID-19 mortality, none of the enrolled diabetic COVID19 patients on statins died because of COVID-19 infection.

Our recent investigations demonstrated that in addition to their anti-inflammatory actions, statins inhibit tissue damage by reducing extracellular matrix synthesis in airway mesenchymal cells [81, 82]. Several studies have shown beneficial effects of statins in the treatment and recovery of patients with idiopathic pulmonary fibrosis and implied that statins improve lung function by acting on fibrosis mediators [83, 84]. In addition, statins reduce mortality in these patients and those with interstitial lung disease [85].

## Conclusion

Overall, we propose that statin use can potentially protect SARS-CoV-2-induced tissue damage and improve lung function in COVID-19 patients via different pleiotropic effects. These effects include targeting the SARS-CoV-2-induced inflammation and cytokine storm, inhibition of cellular trafficking via targeting the autophagy pathway, mediating ACE2 expression, and decreasing extracellular matrix biosynthesis and scar formation in COVID-19 patients. Our current work may have some different results than those reported in the study in China by Zhang et al. [14], likely because of differences in the number (limitation of our current study) and genetic background of the patients involved. Although we could not demonstrate a significant association between statin use and a reduction in COVID19 mortality, our results are promising and clinically relevant and warrant the need for prospective randomized controlled trials and extensive retrospective studies in large and diverse patient populations to further evaluate the potential beneficial therapeutic effects of statin treatment on clinical symptoms and mortality rates associated with COVID-19. We believe that the use of statins could represent a strong adjuvant/adjunct therapeutic strategy in addition to other (antiviral) therapies currently used against COVID-19. The results of our retrospective study provide evidence that using statins (40 mg daily) might render a better clinical outcome for COVID-19 patients. We speculate that long-term usage of statins prior to infection might be most therapeutically effective.

## Data Availability

All data of the manuscript will be provided upon reasonable request and approval by the ethics committee.
